# Association between use of transdermal tulobuterol and short-term outcomes in patients with stroke and underlying chronic obstructive pulmonary disease: A retrospective cohort study

**DOI:** 10.1097/MD.0000000000035032

**Published:** 2023-09-22

**Authors:** Yuichiro Matsuo, Taisuke Jo, Kanako Makito, Hiroki Matsui, Kiyohide Fushimi, Hideo Yasunaga

**Affiliations:** a The Department of Clinical Epidemiology and Health Economics, School of Public Health, The University of Tokyo, Bunkyo-ku, Tokyo, Japan; b The Department of Health Services Research, The University of Tokyo, Bunkyo-ku, Tokyo, Japan; c The Department of Biostatistics, School of Public Health, The University of Tokyo, Bunkyo-ku, Tokyo, Japan; d Department of Health Policy and Informatics, Graduate School of Medical and Dental Sciences, Tokyo Medical and Dental University, Bunkyo-ku, Tokyo, Japan.

**Keywords:** chronic obstructive pulmonary disease, stroke, tulobuterol

## Abstract

Transdermal tulobuterol, a long-acting beta agonist in a transdermal form, is available in some countries, including Japan, Korea, and China. It may provide an alternative treatment option for the management of chronic obstructive pulmonary disease (COPD) in patients who are unable to effectively use inhalers, such as those with acute stroke. This study examined the short-term outcomes of transdermal tulobuterol in patients hospitalized with acute stroke and underlying COPD. Using the Diagnosis Procedure Combination database, a national inpatient database in Japan, we identified patients with stroke and underlying COPD who were hospitalized between July 2010 and March 2021. We performed propensity-score overlap weighting to compare in-hospital death, COPD exacerbation, pneumonia, and cardiac complications between patients who initiated transdermal tulobuterol within 2 days of admission and those who did not use it during hospitalization. Of the 1878 eligible patients, 189 received transdermal tulobuterol within 2 days of admission. After adjusting for baseline variables, transdermal tulobuterol was not associated with a reduction in in-hospital death (18.3% vs 16.1%; odds ratio, 1.17; 95% confidence interval, 0.72–1.90). Additionally, we observed no significant difference in COPD exacerbation, pneumonia, and cardiac complications between both groups. Transdermal tulobuterol was not associated with improving short-term outcomes in patients with acute stroke and underlying COPD. Our study does not support the routine use of transdermal tulobuterol in this patient group. However, further research investigating the long-term efficacy of transdermal tulobuterol in patients with stroke and underlying COPD could help establish its role for the management of these patients.

## 1. Introduction

Stroke shares several risk factors with chronic obstructive pulmonary disease (COPD), including age and smoking. Additionally, patients with COPD were reportedly at a higher risk of developing stroke,^[1]^ and patients with stroke and underlying COPD were at a higher risk of in-hospital complications and mortality than those without COPD.^[2–4]^

The mainstream therapy for stable-state COPD is a combination of inhalers depending on the severity of the disease.^[5]^ Inhalers have been demonstrated to improve COPD outcomes, including improving respiratory symptoms and quality of life, reducing the frequency of acute exacerbations, and reducing mortality.^[6–8]^ However, the effective use of inhalers requires appropriate inhaler techniques. Patients with acute stroke may be unable to use inhaler devices effectively and continue their regular treatment for COPD due to cognitive or functional impairment. For example, a previous study showed 17% of patients with COPD in nursing homes received no respiratory medications, and 39% of patients with cognitive impairment received daily short-acting beta agonist monotherapy through nebulizers,^[9]^ which is not recommended in guidelines. Such non-adherence to the proper treatment for COPD increases the frequency of acute exacerbations^[10]^ and mortality.^[11]^ Additionally, discontinuing COPD treatment may worsen outcomes in patients with COPD who develop acute stroke.

A long-acting beta agonist (LABA) in transdermal form (transdermal tulobuterol) is available in some countries including Japan, Korea, and China.^[12]^ Transdermal tulobuterol can be applied once daily through a skin patch, eliminating the need for inhaler techniques. Therefore, it may be a suitable option for managing patients with stroke with underlying COPD. However, evidence on the efficacy of transdermal tulobuterol for patients with COPD is limited. Some studies have demonstrated that transdermal tulobuterol improved respiratory symptoms,^[13]^ quality of life,^[14]^ and respiratory function from baseline^[15]^ in stable COPD outpatients. Nevertheless, the optimal treatment option for these patients would be inhaler devices. To our knowledge, no study has examined the effectiveness of transdermal tulobuterol in patients with stroke and underlying COPD.

This study aimed to examine whether transdermal tulobuterol could improve outcomes in patients with stroke patients and underlying COPD.

## 2. Methods

### 2.1. Data source

For this retrospective cohort study, we used a nationwide inpatient database in Japan—the Diagnosis Procedure Combination database.^[16]^ This database contains administrative claim data and detailed clinical information on approximately 8 million inpatient admissions annually from approximately 1000 hospitals in Japan. The data includes: the unique identifier of each hospital, patient age, sex, height, weight, main diagnosis, comorbidities at admission, complications after admission, medical procedures and medication administered during hospitalization, modified Rankin scale scores before admission, and at discharge, level of consciousness rated by the Japan coma scale at admission and discharge, activity of daily living scores based on the Barthel index (BI) score at admission and discharge, pre-hospital ambulance use, and admission to stroke care unit or intensive care unit. Additionally, the diagnoses, comorbidities, and complications are coded in the database using the international classification of disease and related health problems 10th revision (ICD-10) codes.

This study was approved by the Institutional Review Board of the University of Tokyo, and written informed consent was waived due to the anonymous nature of the data.

### 2.2. Patient selection

We analyzed data between July 2010 and March 2021 for patients hospitalized with a main diagnosis of ischemic or hemorrhagic stroke (ICD-10 codes: I63 or I61). We restricted the patient cohort to their first hospitalization for stroke during the study period. To identify patients with underlying COPD, we selected those diagnosed with COPD (ICD-10 codes: J41, J42, J43, or J44) during their previous hospitalization or who had a record of COPD listed as a comorbidity during their stroke hospitalization.

Exclusion criteria included patients who: did not have a history of hospitalization within the year before hospitalization for stroke or had not used either inhaler devices or transdermal tulobuterol during their previous hospitalizations; were <40 years at admission; died within 2 days of admission; started using inhaler devices for COPD (long-acting muscarinic antagonists [LAMA], LABA, inhaled corticosteroids [ICS], or combination drugs) within 2 days of admission; experienced COPD exacerbation within 2 days of admission; initiated transdermal tulobuterol on the third day or later during hospitalization; and had a record of COPD or pneumonia as a main diagnosis of the admission.

### 2.3. Transdermal tulobuterol use

We compared the outcomes between patients who used transdermal tulobuterol within 2 days of admission and those who did not use it during their hospitalization. Additionally, we excluded patients who started using transdermal tulobuterol on the third day or later during hospitalization. Notably, since transdermal tulobuterol is occasionally used to treat symptoms of COPD exacerbation, it is difficult to distinguish whether patients who used it later in the course of hospitalization were in a stable state of COPD or experiencing COPD exacerbation.

### 2.4. Outcome measures

The primary outcome was death during hospitalization. Secondary outcomes were COPD exacerbation, pneumonia, and cardiac complications during hospitalization.

COPD exacerbations were defined by the use of systemic corticosteroids in a dose ≥20 mg/day of prednisone. Additionally, pneumonia was defined by the occurrence of bacterial or viral pneumonia or bronchitis as complications after admission. Lastly, cardiac complications were defined by the occurrence of either ischemic heart disease, heart failure, or arrhythmia during hospitalization (See Supplementary Table 1, http://links.lww.com/MD/J842 for details).

### 2.5. Statistical analysis

Continuous variables are presented as means with standard deviations, and categorical variables are presented as numbers with percentages. Missing values were handled by considering them as a distinct category for each variable.

We conducted propensity score overlap weighting^[17]^ to adjust for differences in baseline variables between the transdermal tulobuterol and the control groups. First, we estimated propensity scores using a logistic regression model with the following independent variables: age, sex, body mass index, smoking history, modified Rankin scale scores before admission, level of consciousness rated by Japan coma scale at admission, BI score at admission, the interval from stroke occurrence to admission, pre-hospital ambulance use, stroke care unit or intensive care unit admission, hospital volume (the average annual number of admissions for patients with ischemic or hemorrhagic stroke), the initial diagnosis of ischemic or hemorrhagic stroke, COPD exacerbation within the previous year, asthma exacerbation within the previous year, cardiac disease hospitalization within the previous year, inhaler use (LAMA, LABA, or ICS) in previous hospitalizations, transdermal tulobuterol use in the previous hospitalization, and comorbidities (asthma, diabetes mellitus, hypertension, dyslipidemia, dementia, Parkinson disease, heart failure, ischemic heart disease, atrial fibrillation or atrial flutter, and cancer). Additionally, we included the initiation of the following treatments within 2 days of admission as independent variables: systemic corticosteroid in a dose <20 mg/day of prednisone, theophylline, short-acting beta agonist through nebulizers, tissue plasminogen activators, intubation, and gastric tube or gastrostomy (See Supplementary Table 2, http://links.lww.com/MD/J843 for details). Second, we conducted overlap weighting using propensity scores. In this method, patients in the treatment group are weighted by the probability of not receiving treatment (1-propensity score), and those in the control group are weighted by the probability of receiving treatment (propensity score). Patients whose characteristics are compatible with either treatment (overlap population) contribute relatively more to the final results than do those who are always treated (propensity score near 1) or never treated (propensity score near 0). Overlap weighting methods can estimate the treatment effect in the population with the most treatment equipoise.^[17]^ Third, we calculated standardized mean differences between the groups for each dependent variable. An absolute standardized mean difference exceeding 10% indicated a significant imbalance. Lastly, we conducted generalized linear regression analyses for the outcomes to calculate the odds ratio (OR) and 95% confidence intervals (CI) for the transdermal tulobuterol group with reference to the control group. Robust standard errors were used to calculate the 95% CI.

We performed a subgroup analysis of the outcomes by limiting the analysis to patients with a BI score of <60 at admission, indicating severe neurologic dysfunction. Since patients with severe neurologic dysfunction are less likely to effectively use inhaler devices during hospitalization, this subgroup allowed for a more direct comparison of the treatment effect of transdermal tulobuterol with no treatment. Statistical analysis was conducted using STATA/MP, Version 17 (StataCorp LLC, College Station, TX).

## 3. Results

**Figure 1.**
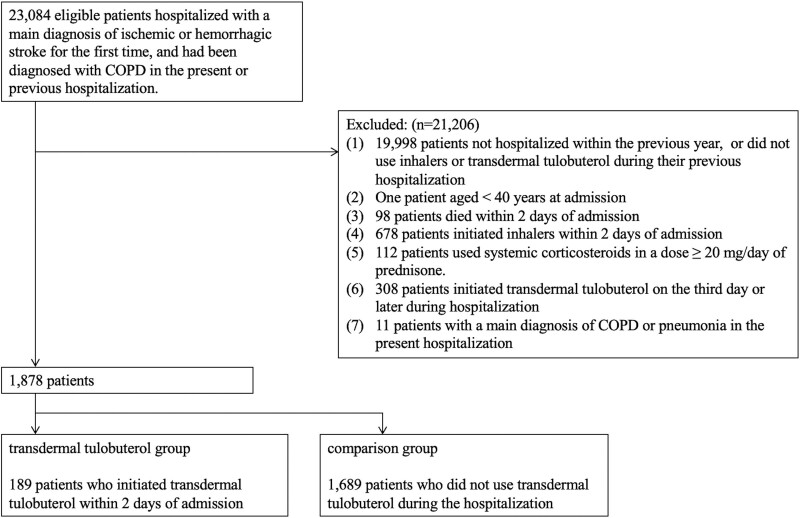
Flowchart of selection of study participants. COPD = chronic obstructive pulmonary disease.

We identified 23,084 initial stroke admissions of patients diagnosed with COPD in the present or previous hospitalization (Fig. 1). After applying the exclusion criteria, our study cohort comprised 1878 patients, divided into the transdermal tulobuterol (n = 189) and control groups (n = 1689). All patients were followed up until discharge from the hospital, wherein no loss to follow-up was reported.

Table 1 presents the baseline characteristics of the patients before and after propensity-score overlap weighting. Compared with the control group, patients in the transdermal tulobuterol group were characterized by older age, a higher proportion of females, lower frequency of previous inhaler use during hospitalization, and higher frequency of previous transdermal tulobuterol use during hospitalization. Furthermore, they had a higher likelihood of receiving concomitant theophylline therapy and a lower likelihood of initiating inhaler treatment on the third day or later during hospitalization than the control group. After the overlap weighting, the patient backgrounds were well balanced between the transdermal tulobuterol and control groups.

**Table 1:** Baseline characteristics before and after propensity-score overlap weighting in the transdermal tulobuterol and control group among patients hospitalized with stroke and underlying COPD.

	Unweighted			Propensity-score overlap weighted		
	Transdermal tulobuterol n = 189	Control n = 1,689	SMD	Transdermal tulobuterol	Control	SMD
Age mean (SD)	81.3 (8.01)	78.3 (8.48)	0.367	81.1	81.1	0.000
Sex			0.315			0.000
Male	135 (71.4)	1425 (84.4)		72.0	72.0	
Body mass index (kg/m^2^)			−0.034			0.000
<18.5	54 (28.6)	430 (25.5)		27.2	27.2	
18.5–25	102 (54.0)	953 (56.4)		55.1	55.1	
≥25	23 (12.2)	211 (12.5)		11.4	11.4	
Missing	10 (5.3)	95 (5.6)		6.4	6.4	
Smoking (Brinkman index)			0.274			0.000
<400	14 (7.4)	96 (5.7)		8.1	8.1	
400–800	18 (9.5)	236 (14.0)		10.4	10.4	
≥800	46 (24.3)	613 (36.3)		25.5	25.5	
Missing	111 (58.7)	744 (44.0)		56.0	56.0	
JCS at admission			0.160			0.000
0	68 (36.0)	792 (46.9)		35.5	35.5	
1–3	82 (43.4)	582 (34.5)		40.6	40.6	
10–30	23 (12.2)	198 (11.7)		14.0	14.0	
100–300	16 (8.5)	117 (6.9)		10.0	10.0	
mRS before admission			0.262			0.000
0	35 (18.5)	532 (31.5)		22.0	22.0	
1–2	36 (19.0)	543 (32.1)		20.1	20.1	
3–5	107 (56.6)	532 (31.5)		51.9	51.9	
Missing	11 (5.8)	82 (4.9)		5.3	5.3	
ADL at admission (Barthel index)			0.262			0.000
0	94 (49.7)	583 (34.5)		48.9	48.9	
5–35	46 (24.3)	359 (21.3)		23.6	23.6	
40–55	19 (10.1)	244 (14.4)		9.1	9.1	
60–95	14 (7.4)	202 (12.0)		8.8	8.8	
100	12 (6.3)	261 (15.5)		7.3	7.3	
Missing	4 (2.1)	40 (2.4)		2.4	2.4	
Length from stroke to hospitalization (d)			0.065			0.000
≤3	147 (77.8)	1440 (85.3)		81.9	81.9	
≥4	35 (18.5)	192 (11.4)		14.7	14.7	
Missing	7 (3.7)	57 (3.4)		3.4	3.4	
Ambulance use	106 (56.1)	877 (51.9)	0.082	58.5	58.5	0.000
SCU/ICU use	22 (11.6)	191 (11.3)	0.010	87.1	87.1	0.000
Annual stroke admissions			−0.092			0.000
≤59	23 (12.2)	133 (7.9)		10.8	10.8	
60–249	78 (41.3)	741 (43.9)		41.8	41.8	
≥250	88 (46.6)	815 (48.3)		47.4	47.4	
Ischemic stroke	157 (83.1)	1371 (81.2)	0.050	82.8	82.8	0.000
History of COPD exacerbation			−0.033			0.000
0	171 (90.5)	1516 (89.8)		90.8	90.8	
1	17 (9.0)	158 (9.4)		8.8	8.8	
≥2	1 (0.5)	15 (0.9)		0.4	0.4	
History of asthma exacerbation			0.016			0.000
0	181 (95.8)	1621 (96.0)		96.5	96.5	
1	8 (3.7)	61 (3.6)		3.2	3.2	
≥2	1 (0.5)	7 (0.4)		0.3	0.3	
History of cardiac disease hospitalization			0.012			0.000
0	158 (83.6)	1421 (84.1)		82.5	82.5	
1	23 (12.2)	198 (11.7)		13.0	13.0	
≥2	8 (4.2)	70 (4.1)		4.5	4.5	
Inlaher use in previous hospitalization						
LAMA	51 (27.0)	1063 (62.9)	−0.774	32.3	32.3	0.000
LABA	36 (19.0)	855 (50.6)	−0.700	24.0	24.0	0.000
ICS	28 (14.8)	561 (33.2)	−0.439	18.2	18.2	0.000
transdermal tulobuterol use in previous hospitalization	165 (87.3)	353 (20.9)	1.786	80.4	80.4	0.000
Comorbidities						
Asthma	30 (15.9)	196 (11.6)	0.125	13.4	13.4	0.000
Diabetes mellitus	41 (21.7)	431 (25.5)	−0.091	20.8	20.8	0.000
Hypertension	82 (43.4)	709 (42.0)	0.029	44.1	44.1	0.000
Dyslipidemia	29 (16.3)	265 (15.7)	−0.010	15.5	15.5	0.000
Dementia	25 (13.2)	93 (5.5)	0.267	12.0	12.0	0.000
Parkinson disease	4 (2.1)	9 (0.5)	0.138	1.0	1.0	0.000
Heart failure	38 (20.1)	270 (16.0)	0.107	19.1	19.1	0.000
Ischemic heart disease	22 (11.6)	170 (10.1)	0.050	10.7	10.7	0.000
Atrial fibrillation or atrial flutter	26 (13.8)	332 (19.7)	−0.159	16.6	16.6	0.000
Cancer	24 (12.7)	340 (20.1)	−0.202	14.2	14.2	0.000
Treatment within 2 d of admission						
Systemic corticosteroid use (<20 mg/d of PSL)	15 (7.9)	79 (4.7)	0.137	6.0	6.0	0.000
Theophylline use	21 (11.1)	38 (2.2)	0.360	6.7	6.7	0.000
SABA use	2 (1.1)	8 (0.5)	0.067	0.3	0.3	0.000
t-PA use	10 (5.3)	69 (4.1)	0.057	6.6	6.6	0.000
Intubation	7 (3.7)	59 (3.5)	0.011	3.8	3.8	0.000
Use of gastric tube or gastrostomy	12 (6.3)	83 (4.9)	0.062	6.6	6.6	0.000
Initiation of inhaler devices on the third d or later (not included in the propensity score calculation)	22 (11.6)	475 (28.1)				

Continuous variables are reported as means and standard deviations. Dichotomous variables are reported as numbers and percentages in the unweighted population. After applying overlap weights, without reporting the raw counts, only the percentages are reported. This is because a single individual no longer represents a single data entity due to the weighting process.

ADL = activity of daily living, COPD = chronic obstructive pulmonary disease, ICS = inhaled corticosteroids, ICU = intensive care unit, JCS = Japan coma scale, LABA = long-acting beta agonist, LAMA = long-acting muscarinic antagonist, mRS = modified Rankin scale, PSL = prednisone, SABA = short-acting beta agonist, SCU = stroke care unit, SD = standard deviation, SMD = standard mean difference, t-PA = tissue plasminogen activator.

The outcomes of both groups are presented in Table 2. After adjusting for baseline variables, transdermal tulobuterol was not associated with a reduction in in-hospital death (18.3% vs 16.1%; OR: 1.17; 95% CI: 0.72–1.90). Similarly, we observed no significant differences in the proportion of COPD exacerbation (7.5% vs 5.4%; OR: 1.39; 95% CI: 0.72–2.67), pneumonia (2.2% vs 1.5%; OR: 1.46; 95% CI: 0.44–4.90), or cardiac complications (22.3% vs 21.8%; OR: 1.03; 95% CI: 0.67–1.59) during hospitalization.

**Table 2:** Outcomes of those treated with or without transdermal tulobuterol in the propensity-score overlap weighted population.

	Transdermal tulobuterol	Control	OR (95% CI)	*P*
Primary outcome				
Death	18.3	16.1	1.17 (0.72–1.90)	.526
Secondary outcomes				
COPD exacerbation	7.5	5.4	1.39 (0.72–2.67)	.321
Pneumonia	2.2	1.5	1.46 (0.44–4.90)	.539
Cardiac complications	22.3	21.8	1.03 (0.67–1.59)	.894

Outcomes are presented as percentages.

CI = confidence interval, COPD = chronic obstructive pulmonary disease, OR = odds ratio.

Subgroup analysis limited to patients with a BI score of <60 on admission showed similar results—use of transdermal tulobuterol was not associated with a reduction in death (Table 3).

**Table 3:** Outcomes of patients with Barthel index <60 at admission treated with or without transdermal tulobuterol in the propensity-score overlap weighted population.

	Transdermal tulobuterol	Control	OR (95% CI)	*P*
Primary outcome				
Death	20.7	18.7	1.13 (0.68–1.89)	.630
Secondary outcomes				
COPD exacerbation	6.5	5.8	1.14 (0.50–2.58)	.762
Pneumonia	2.5	1.8	1.46 (0.42–5.02)	.549
Cardiac complications	26.0	23.2	1.17 (0.73–1.86)	.517

Outcomes are presented as percentages.

CI = confidence interval, COPD = chronic obstructive pulmonary disease, OR = odds ratio.

## 4. Discussion

In this retrospective cohort study using a nationwide inpatient database in Japan, we found no significant difference in in-hospital death, COPD exacerbation, pneumonia, or cardiac complications between the users and non-users of transdermal tulobuterol in patients with acute stroke and underlying COPD. This result remained consistent when the study population was restricted to patients with severe neurologic dysfunction, who are less likely to be able to use inhaler devices.

Previous studies demonstrated that transdermal tulobuterol improved respiratory symptoms^[13]^ and respiratory function^[15]^ from baseline. Moreover, intensive rehabilitation after stroke was associated with a decrease in long-term mortality.^[18]^ Based on these findings, we hypothesized that transdermal tulobuterol use in patients with acute stroke would lead to effective rehabilitation by ameliorating respiratory symptoms, resulting in lower mortality rates. However, our findings did not support this hypothesis. The limited duration of acute hospitalization for stroke may have contributed to the limited effectiveness of transdermal tulobuterol in promoting rehabilitation and reducing mortality.

Although inhaled LABA monotherapy was associated with a reduction in COPD exacerbation,^[7]^ transdermal tulobuterol did not reduce COPD exacerbation or pneumonia during hospitalization; this may be due to the short study period. Furthermore, although transdermal tulobuterol is designed to maintain a stable and effective serum concentration of tulobuterol throughout the day,^[12]^ its clinical effect may differ from that of inhaler devices, which directly administer drugs into the airways. Another possible reason is the less frequent use of inhaler devices during hospitalization in the transdermal tulobuterol group compared with the control group (11.6% vs 28.1%); while patients in the control group resumed their recommended inhaler treatment when they could handle the devices, patients in the transdermal tulobuterol group may have continued using transdermal tulobuterol only. Additionally, patient-specific guideline-recommended combination therapy of LABA, LAMA, and ICS can be administered through inhaler devices; however, with transdermal tulobuterol, only LABA can be administered. This may have led to the insignificant association of transdermal tulobuterol with COPD exacerbation and pneumonia.

In the present study, transdermal tulobuterol was not associated with increased adverse cardiac outcomes. A previous study indicated that the initiation of inhaled LABA was linked to a rise in cardiovascular events.^[19]^ Since transdermal tulobuterol is administered systemically, the activation of beta-2 adrenergic receptors and resulting sympathetic activation may be more significant than the activation in inhaled LABA. Nevertheless, we observed no increased cardiac complications in our study population.

From these results, transdermal tulobuterol for stable COPD in stroke patients may have no short-term benefit, even among those with severe neurologic dysfunction, compared with no treatment. Additionally, since interruption of inhaler treatment was previously associated with worse COPD outcomes,^[10,11]^ efforts should be first made to continue effective treatment with inhalers, even if transdermal tulobuterol is available. This involves assessing the patient inhaler techniques, selecting appropriate devices based on the patient ability,^[20]^ and support from caregivers. Only after these considerations have been made should transdermal tulobuterol be considered an alternative treatment option for those who cannot use inhaler devices.

Our study has some limitations. First, although we adjusted for measured confounders using overlap weighting, we could not account for unmeasured confounders, such as the baseline severity of COPD by the Global Initiative for Chronic Obstructive Lung Disease stage^[5]^ or respiratory symptoms at baseline. However, we adjusted for a history of COPD exacerbation, which is a strong predictor of experiencing new exacerbations.^[21]^ Second, our definitions for the outcomes of COPD exacerbation and cardiac complications have not been validated. Although several studies have shown the validity of respiratory and cardiovascular diseases as the main diagnosis of hospitalization,^[22,23]^ no studies have examined the validity of the diagnosis of new-onset events that occur after hospitalization. We defined COPD exacerbation as the use of systemic corticosteroid at a dose ≥20 mg/day of prednisone. However, using corticosteroids for other reasons, such as allergic reactions, could not be ruled out and the reported percentage of patients experiencing COPD exacerbation in our results may have not accurately reflected the true percentage. Nevertheless, we suggest that the effect of transdermal tulobuterol on COPD exacerbation has unlikely been significantly biased by this definition since using systematic corticosteroids for reasons besides COPD exacerbation would likely be evenly distributed between the 2 groups; the same explanation holds for the definition of cardiac complications. Third, some patients may have continued taking their prescribed outpatient medications, including inhaler devices, during hospitalization. These drugs would not be recorded as prescription drugs in the database. Lastly, our database does not contain information about respiratory symptoms and quality of life during hospitalization; therefore, we could not assess these outcomes, although they are important clinical outcomes of COPD.

Further research is needed to establish the efficacy of transdermal tulobuterol in patients with stroke and underlying COPD. For example, future studies could assess its long-term efficacy, including mortality and risk of COPD exacerbation, in patients with chronic neurologic dysfunction due to stroke who are permanently unable to use inhalers effectively.

## 5. Conclusions

Administration of transdermal tulobuterol in patients with acute stroke and underlying COPD was not associated with differences in death, COPD exacerbation, pneumonia, or cardiac complications during hospitalization. Before considering transdermal tulobuterol for these patients, efforts should be made to continue effective treatment with inhaler devices selected based on the patient ability.

## Author contributions

**Conceptualization:** Yuichiro Matsuo.

**Data curation:** Kanako Makito, Hiroki Matsui, Kiyohide Fushimi.

**Methodology:** Yuichiro Matsuo, Taisuke Jo.

**Supervision:** Taisuke Jo.

**Writing – original draft:** Yuichiro Matsuo.

**Writing – review & editing:** Yuichiro Matsuo, Taisuke Jo, Hiroki Matsui, Hideo Yasunaga.

## Supplementary Material




