# Supplementary material for: Association between use of transdermal tulobuterol and short-term outcomes in patients with stroke and underlying chronic obstructive pulmonary disease: A retrospective cohort study
Source: Medicine (Baltimore). 2023 Sep 22;102(38):e35032. doi: 10.1097/MD.0000000000035032 (PMC10519481; doi:10.1097/MD.0000000000035032)
Supplement: Supplementary file 1 [file medi-102-e35032-s001.docx]

**Supplementary Table 1**. Definition of outcomes

| **Outcome** | **Definition** |
| --- | --- |
| pneumonia | Presence of either of the following ICD-10 codes indicating bacterial or viral pneumonia or bronchitis as complications after admission: J10, J11, J12, J13, J14, J15, J16, J170, J171, J178, J18, J21, J22, J851 |
| cardiac complications | Presence of either of the following ICD-10 codes indicating angina pectoris, acute myocardial infarction, heart failure, or arrhythmias as complications after admission: I200, I201, I208, I209, I210, I211, I212, I213, I214, I219, I110, I500, I501, I509, I470, I471, I472, I479, I480, I481, 482, I483, I484, I489, I490 OR Presence of either of the following procedure codes indicating percutaneous coronary intervention or coronary artery bypass graft surgery: K546, K547, K548, K549, K550, K551, K552 OR Use of either intravenous diuretics, intravenous inotropic agents, or intravenous antiarrhythmic drugs. |

ICD-10, International Classification of Disease and Related Health Problems 10^th^ Revision
