# Supplementary material for: Association between use of transdermal tulobuterol and short-term outcomes in patients with stroke and underlying chronic obstructive pulmonary disease: A retrospective cohort study
Source: Medicine (Baltimore). 2023 Sep 22;102(38):e35032. doi: 10.1097/MD.0000000000035032 (PMC10519481; doi:10.1097/MD.0000000000035032)
Supplement: Supplementary file 2 [file medi-102-e35032-s002.docx]

**Supplementary Table 2**. Definition of baseline variables

| **Variable** | **Definition** |
| --- | --- |
| COPD exacerbation within the previous year | Previous hospitalization with either of the following ICD-10 codes indicating COPD: J41, J42, J43, J44 AND Main diagnosis of either of the following ICD-10 codes indicating COPD or pneumonia/bronchitis in the previous hospitalization: J41, J42, J43, J44, J10, J11, J12, J13, J14, J15, J16, J170, J171, J178, J18, J21, J22, J851, A481, B012, B052, B250 |
| Asthma exacerbation within the previous year | Previous hospitalization with a main diagnosis coded as either of the following ICD-10 codes indicating asthma: J45, J46 |
| Cardiac disease hospitalization within the previous year | Previous hospitalization with a main diagnosis coded as either of the following ICD-10 codes indicating angina pectoris, acute myocardial infarction, heart failure, or arrhythmias: I200, I201, I208, I209, I210, I211, I212, I213, I214, I219, I110, I500, I501, I509, I470, I471, I472, I479, I480, I481, 482, I483, I484, I489, I490 |
|  |  |
| **Comorbidities** | The presence of the following ICD-10 codes in comorbidities present at admission: |
| Asthma | J45, J46 |
| Diabetes mellitus | E11, E12, E13, E14, E888, E891, N189 |
| Hypertension | I10, I11, I12, I13, I15 |
| Dyslipidemia | E78 |
| Dementia | F00, F01, F02, F03, F051, G30 |
| Parkinson’s disease | G20, G21, G22 |
| Heart failure | I110, I50 |
| Ischemic heart disease | I20, I21, I252 |
| Atrial fibrillation or atrial flutter | I48 |
| Cancer | C00-C14, C15-C26, C30-C39, C40-C41, C43-C44, C45-C49, C50, C51-C58, C60-C63, C64-C68, C69-C72, C73-C75, C76-C80, C81-C96, C97, D00-D09 |

COPD, chronic obstructive pulmonary disease; ICD-10, International Classification of Disease and Related Health Problems 10^th^ Revision
